# The DAXX co-repressor is directly recruited to active regulatory elements genome-wide to regulate autophagy programs in a model of human prostate cancer

**DOI:** 10.18632/oncoscience.152

**Published:** 2015-04-17

**Authors:** Lorena A. Puto, Christopher Benner, Tony Hunter

**Affiliations:** ^1^ Molecular and Cell Biology Laboratory, Salk Institute for Biological Studies, La Jolla, CA, USA; ^2^ Integrative Genomics and Bioinformatics Core, Salk Institute for Biological Studies, La Jolla, CA, USA

**Keywords:** ChIP-Seq, RNA-Seq, prostate cancer, DAXX, DNMT1, autophagy

## Abstract

While carcinoma of the prostate is the second most common cause of cancer death in the US, current methods and markers used to predict prostate cancer (PCa) outcome are inadequate. This study was aimed at understanding the genome-wide binding and regulatory role of the DAXX transcriptional repressor, recently implicated in PCa. ChIP-Seq analysis of genome-wide distribution of DAXX in PC3 cells revealed over 59,000 DAXX binding sites, found at regulatory enhancers and promoters. ChIP-Seq analysis of DNA methyltransferase 1 (DNMT1), which is a key epigenetic partner for DAXX repression, revealed that DNMT1 binding was restricted to a small number of DAXX sites. DNMT1 and DAXX bound close to transcriptional activator motifs. DNMT1 sites were found to be dependent on DAXX for recruitment by analyzing DNMT1 ChIP-Seq following *DAXX* knockdown (K/D), corroborating previous findings that DAXX recruits DNMT1 to repress its target genes. Massively parallel RNA sequencing (RNA-Seq) was used to compare the transcriptomes of WT and *DAXX* K/D PC3 cells. Genes induced by *DAXX* K/D included those involved in autophagy, and DAXX ChIP-Seq peaks were found close to the transcription start sites (TSS) of autophagy genes, implying they are more likely to be regulated by DAXX. In conclusion, DAXX binds active regulatory elements and co-localizes with DNMT1 in the prostate cancer genome. Given DAXX's putative regulatory role in autophagy, future studies may consider DAXX as a candidate marker and therapeutic target for prostate cancer.

## INTRODUCTION

According to the American Cancer Society, Cancer Facts & Figures, 2014, among men, carcinoma of the prostate is the second most common cause of cancer death in the US, with 29,480 deaths per year. Once PCa has metastasized, controlling it is often unsuccessful. Current methods and markers used to predict PCa outcome are inadequate. Discovering a new PCa marker that promises to improve prognostication beyond what is achieved with other reported biomarkers becomes of utmost importance.

Many studies have shown that cell death-modulating protein DAXX functions as a transcriptional repressor [1-4]. DAXX associates with RelB, a transcription factor of the NF-κB family that directly controls the expression of numerous apoptosis- and autophagy-relevant genes, including tumor suppressor protein kinases DAPK1/3 [1;2]. A positive connection between DAPK1 and autophagy/tumor suppression has been established [5-8]. In mouse embryo fibroblasts (MEFs) [2], as well as in the PCa cell lines [unpublished data], DAXX represses expression of these genes via mechanisms that include its binding to DNA methyl transferases (DNMTases) and hypermethylation of the promoter regions of the corresponding genes [2]. DAXX also recruits DNMTases to viral DNA so as to initiate its epigenetic repression [4]. A number of publications have reproducibly shown that DAXX interacts directly with DNMTs and employs DNA methylation as a major mechanism of repression [2-4]. DAXX is also known to repress through additional mechanisms, including recruitment of histone deacetylases (HDACs) to target promoters [2], and histone H3.3 association/regulation [9]. Conversely, *DAPK1*, has been shown to be the target of repression in a number of cancers, including AML [10], breast cancer [11], and PCa [Submitted]. Through its ability to repress tumor suppressors, DAXX would be expected to induce tumor growth or survival. It was indeed found DAXX promotes the *in vivo* tumorigenicity of two human PCa cell lines in a subcutaneous tumor xenograft model via suppression of autophagy [Submitted].

Defects in cell death mechanisms represent one of the six identified cardinal features of cancer [12], and are commonly associated with progression of tumors to refractory, chemoresistant disease [13]. Several anti-apoptotic genes have been discovered that are over-expressed in hormone-refractory prostate cancers [14]. A number of lines of evidence indicate that in a cancer context, like in development (*Daxx*-null genotype is an embryonic lethal condition in which the embryos die by age E9.5 [15], due to global apoptosis), DAXX may have a pro-survival role. In prostate tissues, DAXX staining intensity is stronger in areas surrounding tumor glands compared to areas surrounding normal glands [16]. Moreover, strong positive DAXX staining has been observed in PCa tissues, compared to benign prostatic hyperplasia (BPH) tissues, suggesting that DAXX is overexpressed in PCa [17]. In prostate cells, DAXX levels are increased in various PCa cell lines, relative to nontumorigenic human prostatic epithelial lines [17; Submitted]. Conversely, in cells and tissues, DAPK1 and DAPK3 are downregulated in cancer contexts [18; Submitted]. An inverse correlation has been found between DAXX and DAPK1/3 mRNA expression in a diverse collection of human tumor cell lines and tumor specimens [Submitted], suggesting that DAXX's role as a transcriptional repressor of *DAPK1/3* [2] is broadly relevant to tumor biology and is not restricted to PCa. Nevertheless, where DAXX binds in the PCa genome, or what effect it has on the expression of other genes, has not been determined. We performed ChIP-Seq and RNA-Seq to address the above questions. Our results are in agreement with the deposited data in the Oncomine database [Submitted], and establish that DAXX-DNMT1 association is important in PCa progression.

In conclusion, DAXX can serve as an improved marker and attractive therapeutic target in PCa.

## RESULTS

### DNMT1 peaks overlap with, and are dependent on, DAXX

**Figure 1:**
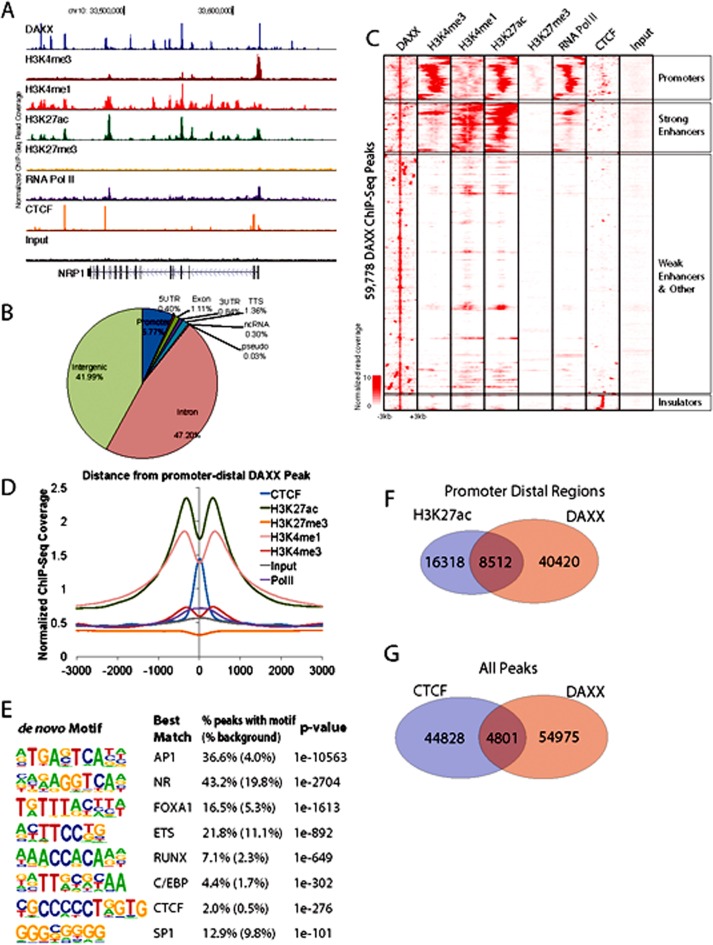
DAXX ChIP-Seq reveals that DAXX binds active regulatory elements **A.** ChIP-Seq binding profile of DAXX in PC3 cells at the NRP1 locus. Previously published (accessed through GEO database repository - NCBI) ChIP-Seq data for chromatin modification H3K4me3, H3K4me1, H3K27ac, H3K27me3, and factors RNA polymerase II and CTCF are shown. **B.** Annotation of 59,778 DAXX ChIP-Seq peaks relative to RefSeq transcript annotations. **C.** Heatmap depicting the ChIP-Seq read density within ±3 kb from DAXX peaks for DAXX and several chromatin marks. Regions were hierarchically clustered to segregate regions with different enrichment patterns. **D.** Enrichment of epigenetic ChIP-Seq experiments relative to promoter-distal DAXX ChIP-Seq peaks. **E.** Results of *de novo* motif enrichment on DAXX ChIP-Seq peaks using HOMER. **F.** Overlap between promoter-distal DAXX and putative enhancers (promoter-distal H3K27ac peaks). Significance of overlap *p*-value < 10^−100^ (binomial test, relative to random genomic distribution). **G.** Overlap between DAXX and CTCF peaks. Significance of overlap *p*-value < 10^−100^ (binomial test, relative to random genomic distribution).

**Figure 2:**
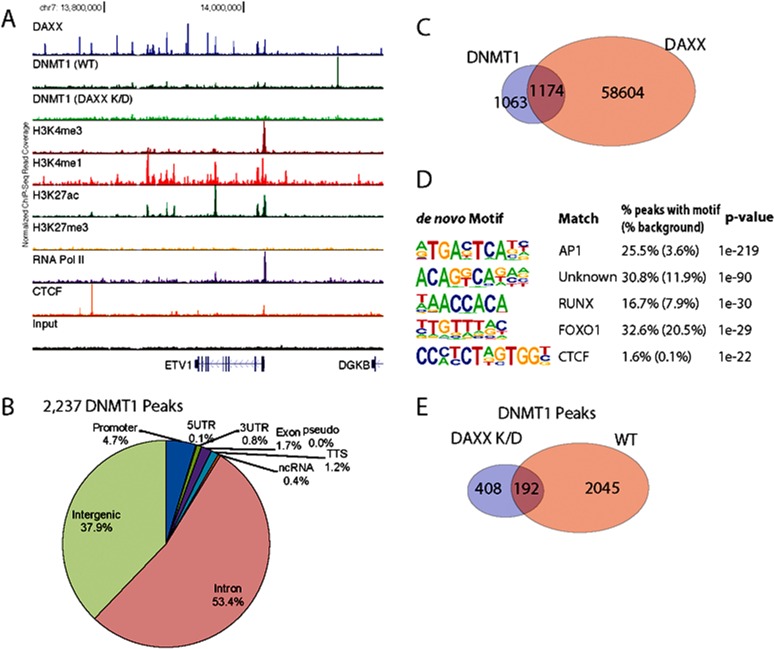
DAXX and DNMT1 ChIP-Seq's reveal that DAXX peaks overlap with DNMT1 peaks **A.** ChIP-Seq binding profile of DAXX, DNMT1, and epigenetic markers in PC3 cells at the ETV1 locus. DNMT1 binding is shown for both WT and *DAXX* K/D conditions **B.** Annotation of 2,237 DNMT1 ChIP-Seq peaks relative to RefSeq transcript annotations. **C.** Overlap between DAXX and DNMT1 peaks. Significance of overlap *p*-value < 10^−100^ (binomial test, relative to random genomic distribution). **D.** Results of *de novo* motif enrichment on DAXX ChIP-Seq peaks using HOMER. **E.** Overlap between DNMT1 peaks found in PC3 WT cells and PC3 *DAXX* K/D cells.

DAXX is a multifaceted protein with a key role in transcriptional repression [1-4], but its genome-wide distribution in PCa via ChIP-Seq has never been investigated. To determine DAXX binding sites in the PCa genome, specifically in the PC3 cell line (derived from bone metastasis of a human PCa case [19]), we generated a stable shRNA knockdown (K/D) PC3 cell line and a control shRNA counterpart [Submitted], and performed a ChIP-Seq study. ChIP-Seq for DAXX was successful, as the strength of the signal in the experimental sample (Figure 1A, top panel) was high compared to the signal from the negative control, the input (Figure 1A, bottom panel). DAXX co-localized with H3K4 and H3K27 histone marks and other factors (RNA Pol II, CTCF) in PC3 cells (Figure 1A). DAXX was found largely at promoter-distal regions, and not on coding regions (Figure 1B). The ~60,000 DAXX peaks found in WT PC3 cells localized to promoters, enhancers, and insulators (Figure 1C). DAXX recruitment to enhancers was at the nucleosome-free region, alongside other transcription factors and co-factors (Figure 1D). De novo motif enrichment showed that DAXX strongly bound to transcriptional activator motifs (Figure 1E), although possibly indirectly to DNA, and likely as part of a complex, due to its lack of DNA binding domains [2]. The motifs included those for ETS transcription factor family, previously shown to interact with DAXX [20]. DAXX bound to a large fraction (34%) of all enhancers in PC3 cells (Figure 1F), and it co-bound with CTCF to nearly 5,000 sites (Figure 1G), consistent with the motif analysis (Figure 1E). Importantly, ChIP-Seq for DNMT1 showed that DNMT1 colocalized with DAXX and was DAXX-dependent (Figure 2A, top three panels). In contrast, the negative control (input, Figure 2A, bottom panel), did not produce specific signals. DNMT1 peaks, similar to DAXX, were found largely at promoter-distal regions (Figure 2B). A large fraction (52%) of DNMT1 peaks were also co-bound by DAXX (Figure 2C), suggesting that DAXX may be required for DNMT1 recruitment. De novo motif analysis of DNMT1 peaks found that AP-1 and an unknown motif were enriched at DNMT1 peaks (Figure 2D). Many of the DNMT1 peaks were lost after *DAXX* knockdown (2,237 DNMT1 peaks in WT (Figure 2C) vs. 600 DNMT1 peaks in *DAXX* K/D (Figure 2E)), indicating that DNMT1 recruitment is almost entirely dependent on DAXX further corroborating previous findings that DAXX recruits DNMT1 to repress its target genes [2].

### Autophagy genes map close to DAXX ChIP-Seq peaks

**Figure 3:**
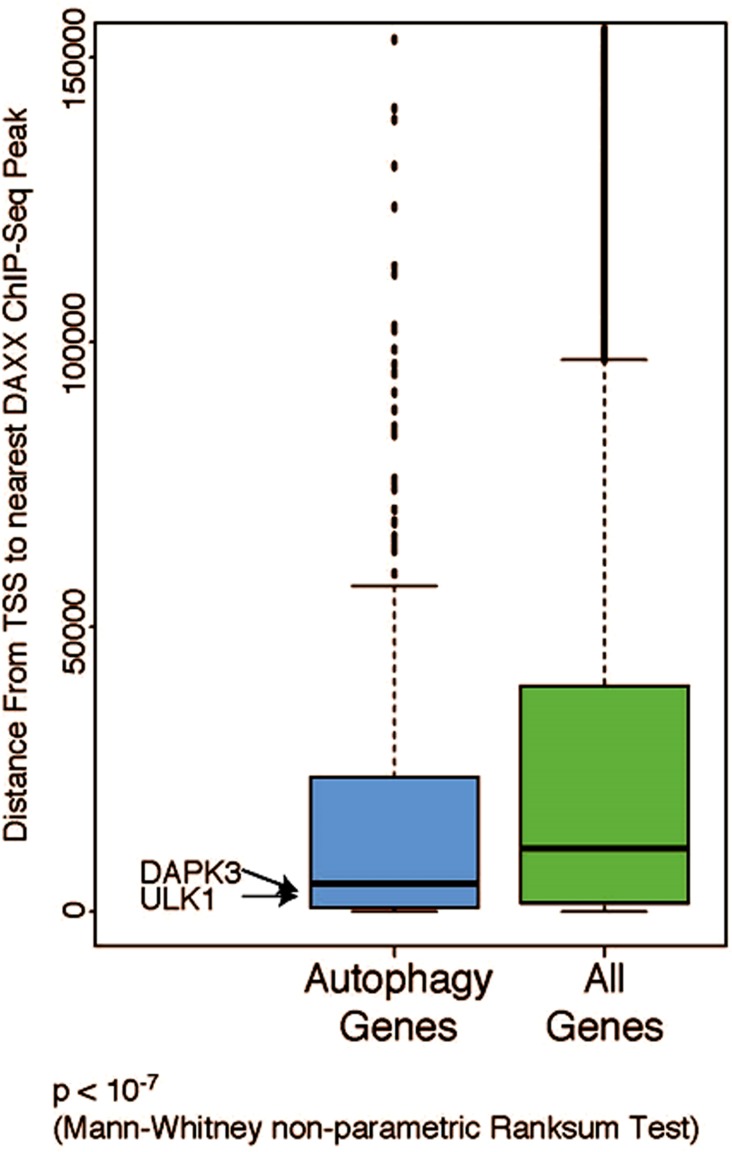
Autophagy genes map closer to DAXX ChIP-Seq peaks than expected Distribution of gene transcription start site (TSS) to the nearest DAXX ChIP-Seq peak was analyzed using HOMER. Regulated gene TSS are normally found closer to the ChIP-Seq peaks than non-regulated peaks. Autophagy genes (http://Tampaku.org/autophagy/list/GeneList) were found to be closer to DAXX ChIP-Seq peaks than all other genes combined, implying that they are more likely to be regulated by DAXX (with the difference being very significant: *p* < 10^−7^). The Y-axis represents distance from TSS to the nearest DAXX ChIP-Seq peak in bp (ULK1: 1,464 bp; DAPK3: 2,401 bp).

Next, the distribution of gene transcription start sites (TSS) nearest to the DAXX ChIP-Seq peaks was analyzed. Regulated gene TSS are normally found closer to the ChIP-Seq peaks than non-regulated peaks [21]. In one study [21], 28% of TSS were located within 5 kb of ChIP-Seq peaks. Autophagy genes, including DAPK3 and ULK1, which were previously shown to be DAXX repression targets [Submitted], were found to be closer to DAXX ChIP-Seq peaks than all other genes combined (Figure 3), implying that they are very likely to be regulated by DAXX (with the difference being highly significant: *p* < 10^−7^).

### DNMT1 levels are reduced in genes upregulated in *DAXX* K/D cells compared to WT cells

**Figure 4:**
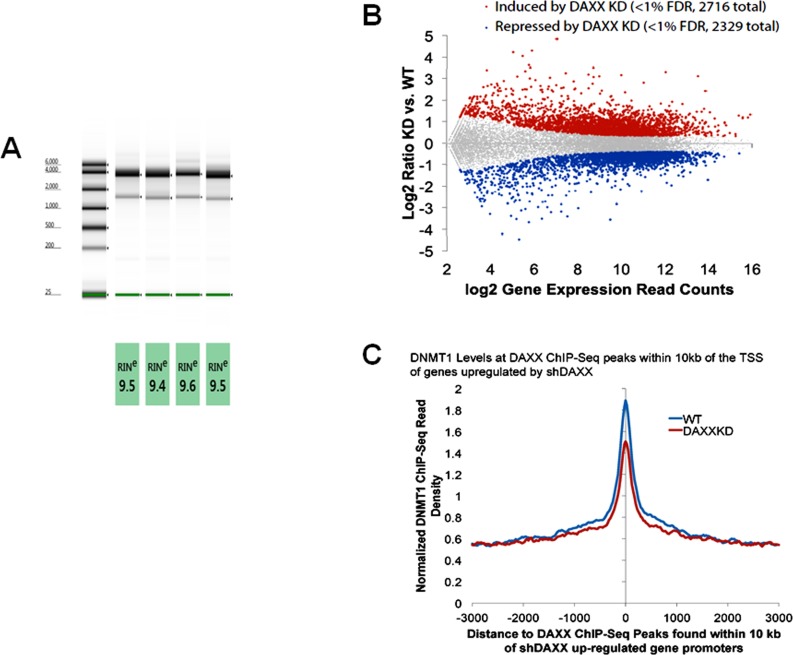
Genes whose expression is increased by *DAXX* knock down in PC3 cells show low levels of DNMT1 association **A.** Total RNA was isolated from PC3 cells as described under Methods. High quality RNA was obtained, as evidenced by high RINs (RNA Integrity Numbers). The first two lanes represent WT samples, whereas lanes 3 and 4 represent *DAXX* K/D samples. **B.** Massively parallel RNA sequencing (RNA-Seq) was used to investigate in an unbiased fashion the expression of different genes, comparing the WT and *DAXX* K/D expression patterns in PC3 cells, using duplicate samples described in **A**.. RNA-Seq reads were aligned to the human genome using STAR. Gene expression was determined using HOMER. 2,716 genes were found to be induced and 2,329 repressed by *DAXX* K/D. Genes induced by *DAXX* K/D included those involved in autophagy. **C.** DNMT1 levels at DAXX ChIP-Seq peaks within 10kb of the TSS of genes upregulated by shDAXX are shown. Genes upregulated in *DAXX* K/D cells have lower DNMT1 levels than those in WT cells, indicating that DNMT1 is employed by DAXX in WT cells to repress these genes.

To identify the genes induced and repressed in WT and *DAXX* K/D cells, RNA-Seq was performed. High quality RNA was first isolated from duplicate samples from each cell line (Figure 4A). RNA-Seq analysis showed that 2,716 genes were increased and 2,329 were decreased in *DAXX* K/D cells compared to control (Figure 4B). Genes induced by *DAXX* K/D included those involved in autophagy (subsequent figures). Next, the ChIP-Seq and RNA-Seq data were co-analyzed. DNMT1 levels at DAXX ChIP-Seq peaks within 10 kbp of the TSS of genes upregulated by shDAXX were determined (Figure 4C). Genes upregulated in *DAXX* K/D cells displayed lower DNMT1 levels than those in WT cells, indicating that DNMT1 is employed by DAXX in WT cells to repress these genes.

### RNA-Seq reveals that DAXX is an autophagy modulator

**Figure 5:**
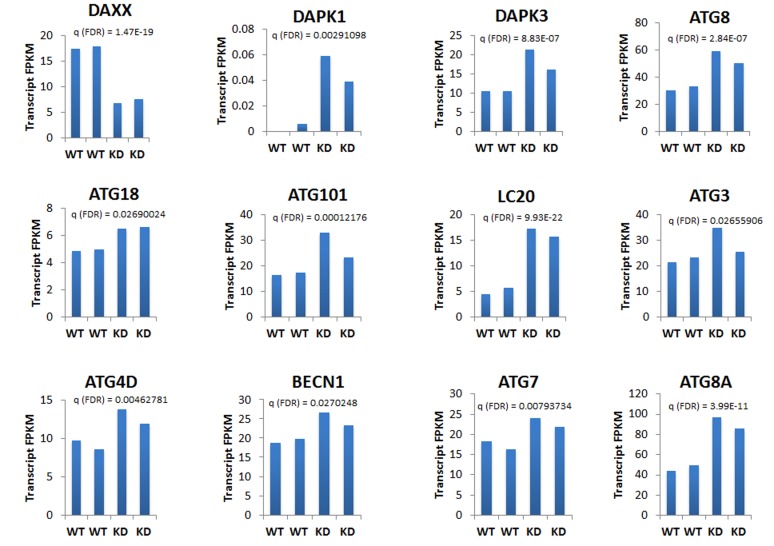
DAXX expression is inversely correlated with the expression of positive regulators of autophagy Using RNA-Seq as described in Figure 4, among autophagy genes, DAXX expression was inversely correlated with expression of positive regulators of autophagy, including DAPK1, DAPK3, ATG8, ATG18, ATG101, LC20, ATG3, ATG4, Beclin1, ATG7, and Deptor (not shown), whereas it was linearly correlated with negative regulators of autophagy mTOR and Raptor (Figure 6). Duplicate samples were used per cell line. The graphs were prepared by determining gene expression using HOMER, and then plotted using Excel.

**Figure 6:**
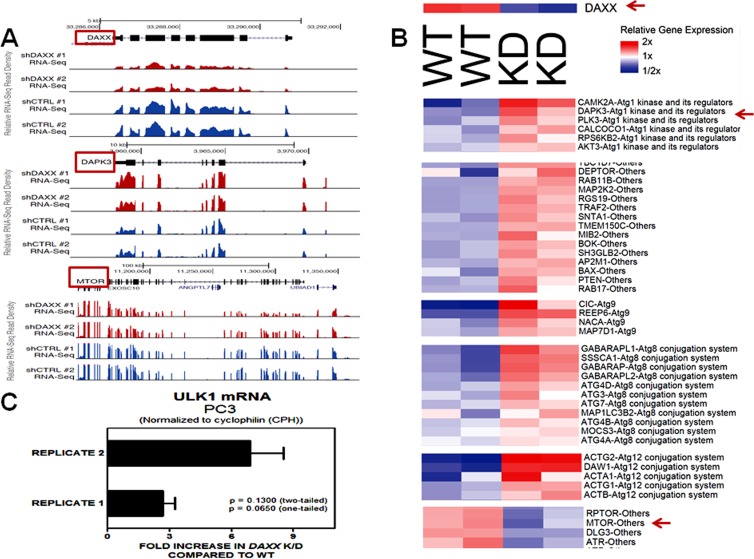
RNA-Seq reveals a connection between DAXX and autophagy regulators Genome browser examples **A.** and a condensed heat map from RNA-Seq experiments **B.** show an inverse correlation between DAXX and DAPK3 and a linear correlation between DAXX and mTOR. Representative values are plotted in Figure 5. Read densities were visualized by preparing normalized BigWig files using HOMER and uploading them to the UCSC Genome Browser **A**., as described under Methods. Visualization of gene expression values as heatmaps **B.** was performed using Java TreeView. Duplicate samples were used per cell line. **C.** Q-PCR analysis was performed using cDNA samples as described under Materials and Methods to determine ULK1 mRNA levels in WT and *DAXX* K/D cells, and compare them to RNA-Seq results. *DAXX* K/D cells show increased levels of ULK1 mRNA compared to WT cells. The variability between replicates was not statistically significant.

Based on ChIP-Seq findings (Figure 3), we focused our analysis on the relationship between DAXX and autophagy genes. To that end, RNA-Seq was utilized, using duplicate samples per cell line (WT and *DAXX* K/D). DAXX expression correlated inversely with expression of positive regulators of autophagy, including DAPK1, DAPK3, ATG8, ATG18, ATG101, LC20, ATG3, ATG4, Beclin1, ATG7 (Figure 5), and Deptor (not shown), whereas it correlated positively with expression of negative regulators of autophagy, mTOR and Raptor (Figure 6A and 6B). DAXX may increase mTOR/mTORC1/Raptor activity by suppressing ULK1 [Submitted], which, through a negative feedback loop, phosphorylates and inhibits Raptor and subsequently inhibits mTORC1 signaling [22]. Through an as of yet unidentified mechanism, DAXX may also affect mTOR and Raptor levels, in addition to their activity. As expected, DAXX RNA expression levels were decreased (65%) in *DAXX* K/D cells (Figure 5, 6A, and 6B). Genome browser examples (Figure 6A) and a condensed heat map from RNA-Seq experiments (Figure 6B) show an inverse correlation between DAXX and DAPK3 and a linear correlation between DAXX and mTOR. Therefore, in conjunction with previous findings [Submitted], DAXX appears to repress autophagy in PCa. Of note, an effect on ULK1, another DAXX repression target [Submitted], was not observed via RNA-Seq. To validate RNA-Seq data on ULK1, we performed Q-PCR using the same RNA that was used for RNA-SEQ. Q-PCR showed that ULK1 mRNA levels were increased 3-7- fold in *DAXX* K/D samples, using the cyclophilin (CPH) internal control gene for normalization (Figure 6C). Although there was variability between replicates, it did not amount to significant difference (Figure 6C). Use of an alternative internal control, (GAPDH), showed a similar fold increase (not shown). The reason for the difference in the fold change in ULK1 RNA levels observed in WT and *DAXX* K/D PC3 cells between the RNA-Seq and Q-PCR analyses is unclear, but we believe that the Q-PCR result is reliable based on the replicates we obtained.

## DISCUSSION

In the current study, utilizing ChIP-Seq, we investigated the genome-wide binding of the DAXX transcriptional repressor and its epigenetic partner DNMT1 in the PC3 PCa cell line, and found that DNMT1 enrichment is dependent on DAXX. Importantly, ChIP-Seq showed that autophagy genes mapped closer to DAXX peaks than all other genes combined, indicating that they are likely to be regulated by DAXX. Additionally, using RNA-Seq, we determined changes in gene expression levels between the wild-type (WT) and *DAXX* knock-down (K/D) PC3 cells. Genes increased by *DAXX* K/D included those involved in autophagy. Related to autophagy genes, ChIP-Seq and RNA-Seq data showed significant overlap. Specifically, among autophagy genes, the presence of DAXX was inversely correlated with the expression of positive regulators of autophagy - DAPK1, DAPK3, ATG8, ATG18, ATG101, LC20, ATG3, ATG4, Beclin1, ATG7, and Deptor, whereas it correlated positively with negative regulators of autophagy – mTOR and Raptor.

The current study provides further clarification on the role of the DAXX transcriptional repressor in prostate tumorigenesis and progression. In many types of cancer, including PCa [23], tumor suppressors, such as DAPK1 and DAPK3, are downregulated, a process that facilitates cancer metastasis. One of the proteins that represses the *DAPK1* and *DAPK3* genes is DAXX [2], and our working hypothesis is that suppression of *DAPKs* by DAXX results in increased prostate tumorigenesis and progression [Submitted]. The goal is to restore the activity of DAPKs in patients with advanced prostate cancer by inhibiting production of DAXX, for example via tissue-specific shRNA delivery, which has already shown potential in cancer therapy [24]. The results of this project may guide current therapeutic efforts to employ tissue-specific shRNA delivery in the treatment of metastatic PCa. This approach would inactivate the DAXX pathway for tumor promotion, and consequently restore the activity of the DAPK1 and DAPK3 tumor suppressors. In combination with this approach, inhibitors of DNA methylation and/or histone deacetylases are expected to relieve repression of DAPK tumor suppressors, whose epigenetic silencing has emerged as a recurring theme from our work and throughout the literature. Restoring the activity of endogenous tumor suppressors is an attractive strategy for controlling malignancy, because it taps into natural pathways for self-defense against cancer.

Our study represents the first to determine DAXX location in the PCa genome, as well as its correlation with the expression of PCa genes. We utilized the PCa cell line PC3, derived from a PCa bone metastasis [19]. An important finding was that DNMT1 ChIP-Seq peaks overlapped with DAXX peaks (Figure 2), and that DNMT1 was dependent on DAXX for recruitment to specific sites in the PCa genome. The DNMT1 number of ChIP-Seq peaks was considerably lower than that of DAXX, but in agreement with other reported ChIP-Seq studies involving DNMT1 [25].

Corroborating our recent findings [Submitted], through ChIP-Seq studies, DAXX was found to be strongly bound to its repression targets - DAPK3 and ULK1 – although DNMT1 was not found to have the same binding affinity for the promoters of these genes (10 kbp promoter length was examined). It is possible that DAXX employs several mechanisms to repress its targets, besides DNA methylation through DNMT1.

Also reinforcing our recent observations [Submitted], both ChIP- and RNA-Seq revealed that DAXX represses positive modulators of autophagy. DAXX expression inversely correlated with DAPK3 (Figure 5, 6A, 6B: RNA-Seq), and ULK1 (Figure 6C: Q-PCR).

Oncomine analyses [Submitted] have shown a significant correlation between elevated DAXX expression and PCa development and progression/metastasis. Larger scale analyses will be needed to correlate expression of DAXX with pathological stage and survival. Future studies will also include immunohistochemistry analyses to confirm the bioinformatics/gene expression analyses. In addition to DAXX, immunohistochemistry studies will include DAPK1 and DAPK3 so as to establish a relationship between DAXX and the DAPK1/3 tumor suppressors. The correlations between DAXX and DAPK1/3 expression will be based on the tumor grade, stage, and disease metastasis. This study design will establish whether (1) DAXX can serve as a marker for early tumor growth or later advanced/metastatic PCa, and (2) high DAXX expression associates with indolent versus lethal PCa.

In conclusion, bound DAXX co-localizes with bound DNMT1 in the prostate cancer genome. DAXX represses autophagy gene expression, presumably also because of its ability to recruit DNMT1, among other mechanisms. Autophagy acts as a tumor suppressor mechanism in PCa [Submitted]. We can envision that DAXX can serve as a precise prognostic marker in PCa and that tissue-specific shRNA delivery targeted against DAXX can be employed in the treatment of metastatic prostate cancer in patients, in particular if we discover, through additional studies, that DAXX is a marker for lethal PCa in humans. Improved knowledge of DAXX and its mechanisms will be helpful for identifying those patients for whom tissue-specific shRNA delivery will be beneficial, thus ensuring that therapeutic intervention is appropriately tailored to each patient's disease.

## MATERIALS AND METHODS

### Cell culture and recombinant lentivirus transduction

The human prostate cancer cell line PC3, which was obtained from Scott Crist (Purdue University), was maintained in Roswell Park Memorial Institute-1640 (RPMI-1640) medium, containing 10% fetal bovine serum, FBS (HyClone), and 1% penicillin/streptomycin plus L-glutamine. For the generation of a stable *DAXX* knock-down (K/D) PC3 line, recombinant lentiviruses targeting *DAXX* (constructed in the lentiviral backbone vector pLKO.1-puro) were purchased from Sigma (Clone ID: NM_001350.x-2410s1c1; Accession Number: NM_001350.3; Region: 3UTR). A non-specific control virus was also purchased (SHC002V: MISSION® Non-Target shRNA Control Transduction Particles).

### Chromatin Immunoprecipitation (ChIP), Deep Sequencing (ChIP-Seq), and RNA Sequencing (RNA-Seq)

ChIP was performed using the Active Motif's ChIP-IT High Sensitivity kit (cat # 53040), starting with the control (WT or non-specific shRNA) and DAXX K/D PC3 cells. The manufacturer's protocol was followed. The following goat polyclonal antibodies from Santa Cruz Biotechnology Inc. were used for ChIP: anti-DAXX (sc-7001) and anti-DNMT1 (sc-10219). A two-step cross-linking procedure (protein-protein & DNA-protein) preceded ChIP as described [26], using disuccinimidyl glutarate (DSG) for protein-protein cross-linking (Thermo Scientific, cat # 20593). For ChIP, anti-DAXX or anti-DNMT1 antibodies were used to collect chromatin bound to the respective antibody, and was followed by deep sequencing (ChIP-Seq) using an Illumina HiSeq 2500 system. Non-immunoprecipitated (input) chromatin, subjected to the same treatment, served as a control. Massively parallel RNA sequencing (RNA-Seq) was used to investigate in an unbiased fashion the expression of different genes, comparing the control and DAXX K/D expression patterns in PC3 cells (duplicate samples). Total RNA was first isolated using NucleoSpin RNA kit (Clontech, cat # 740955). The RNA integrity number (RIN) was determined using The Agilent RNA ScreenTape assay (Agilent 2200 TapeStation, Salk Institute's Next Generation Sequencing core), and high quality RNA was used for RNA-Seq. The RNA-Seq libraries were prepared using Illumina TruSeq RNA v2 non-stranded sample prep kits and sequenced on an Illumina HiSeq 2500 according to the manufacturer's instructions. Genomic data analysis and visualization of ChIP-Seq and RNA-Seq analyses were performed as described below.

### Genomic data analysis and visualization

#### ChIP-Seq Analysis

ChIP-Seq sequencing reads were mapped to the human genome (GRCh37/hg19) using Bowtie2 with default parameters (v2.1.0, [27]). Only reads that mapped to a single unique location were considered for further analysis. Genome browser BedGraph tracks and read density histograms were generated using HOMER [28]. Peak finding and annotation to the nearest RefSeq gene promoter was performed using HOMER (findPeaks program using the “-style factor” option). *De novo* motif discovery was also carried out using HOMER.

#### RNA-Seq Analysis

RNA-Seq reads were aligned to the human genome (GRCh37/hg19) using STAR with default parameters (v2.1.4a, [29]). Only reads that aligned to a single, unique location where kept for further analysis. Read densities were visualized by preparing normalized BigWig files using HOMER and uploading them to the UCSC Genome Browser (http://genome.ucsc.edu/). Gene expression was determined by identifying reads on the appropriate strand overlapping exons defined by RefSeq using HOMER. Differentially expressed genes were then identified using EdgeR [30]. Functional Enrichment with Gene Ontology was performed using DAVID [31]. Visualization of gene expression values as heatmaps was performed using Java TreeView [32].

Both ChIP-Seq and RNA-Seq analyses were performed using Salk Institute's Next Generation Sequencing core facility.

#### Q-PCR analysis

PC3 and PC3 *DAXX* K/D cells were processed using Power SYBR Green Cells-to-Ct Kit (Ambion, Cat # 4402953) to lyse cells, generate cDNA, and perform Q-PCR per the manufacturer's instructions. Applied Biosystems 7900HT Q-PCR instrument was used to amplify the samples, and the analysis was performed using SDSv2.4 software (Life Technologies). All statistical analyses were performed using GraphPad Prism 5. The sequences of the ULK1 and control (GAPDH & CPH) Q-PCR primers are as follows:

ULK1 forward primer: 5′-CAA GAG CAC ACG GAG ATC CTG C-3′;
ULK1 reverse primer: 5′-CCC CAT TCT CGG CTC AGC AGG-3′;
GAPDH forward primer: 5′-ACA TCA AGA AGG TGG TGA AGC AGG-3′;
GAPDH reverse primer: 5′-ACA AAG TGG TCG TTG AGG GCA ATG-3′;
CPH forward primer: 5′-GAC CCA ACA CAA ATG GTT C-3′;
CPH reverse primer: 5′-AGT CAG CAA TGG TGA TCT TC-3′.
